# CSF cytology: core principles, diagnostic pitfalls and emerging digital tools including artificial intelligence and telecytology

**DOI:** 10.1007/s00401-026-03101-7

**Published:** 2026-10-30

**Authors:** Sandra Nay, Franz Felix Konen, Franziska Bachhuber, Alexander Dressel, Deborah Erhart, Andreas Gerritzen, Catharina C. Gross, Andrea Harrer, Andrea Hinger, Piotr Lewczuk, Jan Lewerenz, Michael Khalil, Mirjam Korporal-Kuhnke, Frank Leypoldt, Thiemo M. Möllenkamp, Markus Otto, Astrid Petersmann, Axel Regeniter, Martin Roskos, Klemens Ruprecht, Annette Spreer, Herwig Strik, Marie Süße, Hayrettin Tumani, Manfred Uhr, Manfred Wick, Brigitte Wildemann, Jens Wiltfang, Thomas Zimmermann, Thomas Skripuletz, Stefan Isenmann

**Affiliations:** 1 Department of Neurology, Hannover Medical School, 30625 Hannover, Germany; 2 Department of Neurology, University of Ulm, 89081 Ulm, Germany; 3 Department of Neurology, Medical University Lausitz-Carl Thiem, 03048 Cottbus, Germany; 4 Medical Laboratory Bremen, 28355 Bremen, Germany; 5 Department of Neurology, University Hospital and University Münster, 48149 Münster, Germany; 6 Department of Neurology, Christian-Doppler University Hospital, Paracelsus Medical University, 5020 Salzburg, Austria; 7 Department of Dermatology and Allergology, Paracelsus Medical University, 5020 Salzburg, Austria; 8 Division of Neuroimmunology, Department of Neurology, Heidelberg University Hospital, 69120 Heidelberg, Germany; 9 Department of Neurology, Friedrich-Alexander-University and University Hospital Erlangen-Nürnberg, 91054 Erlangen, Germany; 10 Department of Neurodegeneration Diagnostics, Medical University of Białystok, Białystok, Poland; 11 Department of Biochemical Diagnostics, University Hospital of Białystok, 15-269 Białystok, Poland; 12 Department of Neurology, Medical University of Graz, 8036 Graz, Austria; 13 Institute of Clinical Chemistry and Department of Neurology, University Hospital Schleswig-Holstein and Kiel University, 24105 Kiel, Germany; 14 Department of Neurology, Martin-Luther-University Halle-Wittenberg, 06120 Halle (Saale), Germany; 15 Institute for Clinical Chemistry and Laboratory Medicine, University Medicine Oldenburg, 26129 Oldenburg, Germany; 16 Infectious Disease Serology and Immunology, Medica Medizinische Laboratorien Dr. F. Kaeppeli AG, 8032 Zurich, Switzerland; 17 SYNLAB Holding Deutschland GmbH, 86156 Augsburg, Germany; 18 Department of Neurology, Klinikum Braunschweig, 38126 Brunswick, Germany; 19 Department of Neurology, Sozialstiftung Bamberg, 96049 Bamberg, Germany; 20 Department of Neurology, University Medicine Greifswald, Greifswald, Germany; 21 Department of Psychiatry, Max Planck Institute of Psychiatry, 80804 Munich, Germany; 22 Institute of Laboratory Medicine, LMU University Hospital, LMU Munich, 81377 Munich, Germany; 23 Department of Psychiatry and Psychotherapy, University Medical Center Göttingen, 37075 Göttingen, Germany; 24 Labopart Medical Laboratories, 01127 Dresden, Germany; 25 Department of Neurology, GFO Kliniken Niederrhein, St. Josef Krankenhaus Moers, 47441 Moers, Germany

**Keywords:** Pleocytosis, Cytomorphology, Leptomeningeal disease, Lumbar puncture, Ventriculitis, Digital pathology

## Abstract

Cerebrospinal fluid cytology remains a cornerstone of diagnostic evaluation in infectious, autoimmune, haemorrhagic, and neoplastic disorders of the central nervous system despite advances in neuroimaging, molecular diagnostics, and digital technologies. This narrative review summarises established principles of cerebrospinal fluid cytology, highlights methodological limitations and diagnostic pitfalls, and integrates emerging developments, including automated cell counting, artificial intelligence, and telecytology. Pre-analytical factors are critical determinants of diagnostic accuracy. Delayed sample processing promotes pH-dependent autolysis and substantial cell loss, whereas rapid cytocentrifugation preserves cellular morphology and facilitates reliable assessment. Automated cell counters are increasingly implemented in routine laboratories and offer advantages in settings with limited technical staffing. However, current systems show limited precision in samples with low and slightly elevated cell counts and limited morphological evaluation. Manual chamber counting, particularly in the normal and low-cell-count range, remains the gold standard for quantitative CSF cell assessment, whereas expert microscopic examination remains the reference standard for qualitative cytomorphological assessment. Although the sensitivity of neuroimaging continues to improve, cerebrospinal fluid cytology retains a unique diagnostic role in the detection of infectious and neoplastic conditions and in the evaluation of subarachnoid haemorrhage. Cytological detection of erythrophages, siderophages, and haematoidin crystals can reveal haemorrhage and provide clues to its temporal stage. Overall, cerebrospinal fluid cytology remains a valuable diagnostic tool when integrated with clinical, laboratory, microbiological, immunological, and imaging findings. Careful recognition of common pitfalls is essential. We recommend rapid cerebrospinal fluid processing, routine preparation of cytospin-based stained slides with expert microscopic review, and the implementation of automated or digital methods only within clearly validated workflows.

**Supplementary Information:**

The online version contains supplementary material available at https://doi.org/10.1007/s00401-026-03101-7.

## Introduction: fundamentals of cerebrospinal-fluid differential cell analysis

Cerebrospinal fluid (CSF) is primarily produced by the choroid plexus of the lateral ventricles and circulates through the ventricular system, subarachnoid space, and the central canal of the spinal cord. It acts both as a mechanical cushion for the central nervous system (CNS) and a medium for transporting signalling molecules, metabolites, and proteins. Daily CSF production amounts to approximately 500 mL, while the total volume present is 150–170 mL. Therefore, the CSF is replaced every five to seven hours [125]. For diagnostic purposes, CSF is typically collected via lumbar puncture at the L3/4 or L4/5 intervertebral space. Suboccipital or lateral cervical puncture is performed only rarely and has largely been replaced by lumbar and ventricular CSF access. In selected scenarios, ventricular CSF may be obtained, usually via external drainage systems. Under physiological conditions, CSF is macroscopically clear, colourless, and nearly acellular. Adequate CSF volumes are essential to ensure diagnostic accuracy, particularly during the initial diagnostic work-up. In addition to cell counting and microscopy, standard analysis should include CSF protein, glucose, and lactate levels, as well as albumin and immunoglobulin quotients [128]. In some regions, particularly in German-speaking countries, these parameters are routinely interpreted using CSF/serum quotient diagrams [102, 128]. While this cytology-focused review will not elaborate on these parameters, it should be noted that collecting and integrating them alongside the other results and clinical findings is essential for preparing a cumulative integrated CSF report. Importantly, cytological assessment of at least one stained slide is crucial [128].

Table 1 summarises key practical questions that guide CSF cytology in the diagnostic work-up of CNS pathology which are addressed throughout this review.

**Table 1:** Key questions for cerebrospinal fluid cytology in the diagnostic work-up of nervous system pathology

Key questions	References
1. What is the gold standard for CSF cell diagnostics? Manual chamber counting remains the gold standard for quantitative CSF cell assessment (particularly in the normal and low-cell-count range), while automated analysers may be used as a first-line approach to ensure availability and rapid turnaround. A threshold of <30 cells/µL may be used as a pragmatic criterion for manual confirmation when no lower, locally validated threshold has been established Differential cell count and morphological assessment must be performed by an experienced cytologist using microscopy of cytocentrifuge-prepared slides, ideally stained with Pappenheim (May-Grünwald-Giemsa). Automated differentiation may support screening but does not replace dedicated cytological evaluation	[128, 134, 136]
2. Is preparation of cytological slides redundant in samples with normal CSF cell count? No. Relevant pathological findings (e.g. malignant cells, activated lymphocytes, macrophage inclusions) may occur despite normal total cell counts Cytology therefore provides diagnostic information beyond quantitative cell counts alone	[11, 118]
3. Are pre-analytical factors relevant for interpretation of CSF cell count and cytomorphology findings? Yes. Delay between lumbar puncture and analysis, storage temperature, blood contamination, and insufficient sample volume significantly affect cell integrity and morphology Processing CSF samples within 1 to 2 h is considered critical Particularly on first work-up, a minimum of 5 mL—preferably 10–15 mL—should be collected for comprehensive CSF diagnostics At least 2–3 mL should be available for cytology alone, while higher volumes are recommended when malignancy is suspected	[17, 29, 77, 97, 99, 115, 128]
4. Does blood contamination affect the diagnostic accuracy of CSF cytology? Yes. Blood admixture may cause pseudopleocytosis and complicates interpretation of differential cell counts	[9, 56]
5. Does ventricular cerebrospinal fluid differ cytologically from lumbar CSF? Yes. Ventricular CSF typically shows lower cell counts compared with lumbar CSF Cellular composition may differ due to rostrocaudal gradients and local pathology (e.g. intraventricular haemorrhage, shunt-associated inflammation) Reference values derived from lumbar CSF are therefore not directly applicable to ventricular samples	[26, 54, 95]
6. Do cytological reference values and interpretations established in adults also apply for paediatric CSF samples? No. Neonates and young infants physiologically exhibit higher CSF white blood cell counts compared to adults Age-specific reference ranges are mandatory to avoid misclassification of physiological findings as pathological Cytomorphological interpretation must take developmental aspects into account	[59, 81, 120, 144]
7. Can CSF cytology be altered by intrathecal therapies? Yes. Intrathecal therapies (e.g. nusinersen, tofersen, corticosteroids) and systemic treatments (e.g. IVIG) may induce sterile pleocytosis or characteristic macrophage inclusions These changes are often asymptomatic and must be recognized as treatment-related to avoid misdiagnosis	[40, 58, 65, 119, 126, 137]

### Literature search and review approach

This article was conceived as a narrative, expert-driven review rather than a systematic review. Literature was identified through iterative, topic-specific database searches throughout manuscript preparation, supplemented by reference tracing and consultation of relevant textbooks, monographs, and cytology atlases. Given the aim of integrating the historical development of CSF cytology with current diagnostic practice and emerging technologies, no predefined publication period or fixed search string was applied. Both historical and contemporary publications were considered. Literature was selected according to its relevance to the scope of the review and its contribution to current diagnostic practice or historical context. Where findings or recommendations differed between publications, these discrepancies were retained and discussed rather than resolved by formal evidence weighting, with current guidelines, reviews, and primary studies used to contextualize areas of uncertainty. No geographical restrictions were applied, and evidence from international guidelines and primary studies was considered where relevant.

Concerning Additional Table 1: To update the evidence concerning manual vs. automated CSF cell quantification summarised by [134] PubMed/MEDLINE and the Cochrane Library/CENTRAL were searched on 19 September 2026 for studies published between 1 January 2019 and 19 September 2026. The targeted search combined terms for cerebrospinal fluid/CSF with terms relating to automated cell counting or body-fluid analysers and manual counting methods, including counting chamber/haemocytometer-based approaches. The database search identified 373 records (PubMed/MEDLINE, *n* = 341; Cochrane Library/CENTRAL, *n* = 32). After removal of four duplicates, 355 of 369 records were excluded during title and abstract screening. Fourteen full-text articles were assessed, of which 11 were included. Citation screening identified six additional full-text articles, of which four were eligible. Across database and citation screening, five full-text articles were excluded: one because it had already been included in Waldrop et al., two because the primary quantitative comparison was between automated platforms without an eligible manual quantitative reference, one because manual microscopy was restricted to differential assessment without an appropriate manual WBC/TNC reference comparison, and one because CSF-specific quantitative results were not separately extractable. In total, 15 studies were included in Supplementary Table S1.

### CSF cytology—history and fundamentals

Quincke reported on the first transcutaneous lumbar puncture in the late nineteenth century and continued to study CSF in the ensuing years, including CSF pressure, chemical components, cytology, and findings in various pathologies [98]. Much of the early development in CSF diagnostics took place in German-speaking countries, such as development of the Fuchs-Rosenthal chamber [35] and the so-called panoptic Pappenheim staining [90]. In the early days, cell yield was sparse and cellular appearance often disrupted, making morphological diagnosis difficult, as illustrated in the first CSF atlases [53, 101].

It was the introduction of the sedimentation chamber by Sayk [108] in the 1950 s that paved the way to more reliable analysis of CSF cells. Thus, illustrated works with good-quality cell appearance appeared in the 1960 s and 1970 s from Jena and Halle, Germany. Sayk provided a first photographic atlas [109], followed by Schmidt [111], who conducted extensive work on CSF cytology of inflammatory processes, tissue regeneration, phagocytosis, and atypical cells. With the arrival of the gentle centrifugation technique using a cytocentrifuge, CSF cytology became more feasible, and further works followed by independent authors that were primarily published in English [64, 85]. In parallel, American works appeared independently [44, 106], with limited cross-referencing.

In contrast to the substantial advances in CSF biochemistry and biomarker research, the fundamental morphological features and staining characteristics of cells in the CSF have remained stable over time. Rather than changes in morphology, historical controversies in CSF cytology mainly concerned the interpretation and classification of cell populations, including lymphocyte subtypes, macrophage activation states, and the distinction between reactive and neoplastic cells. In parallel, evolving tumour nomenclature and molecular classification systems necessitate cautious interpretation of older cytological literature.

For readers interested in a comprehensive and practice-oriented approach to CSF cytology, several dedicated atlases and textbooks are currently available [74, 141]. Kluge and co-authors provided an up-to-date CSF-cytology atlas in German that set the standards for approximately 20 years [61], and—uniquely—was translated into English [62] and supplemented by DVDs for teaching and learning purposes in both languages. More recently, a comprehensive work on all aspects of clinical CSF diagnostics was updated and now reflects the state-of-the-art practice [141]. Some of the authors of the present article contributed to this work, which is disclosed here for reasons of transparency. The chapter on CSF cytology [48], and an integrated case atlas [47] illustrate typical CSF cytology findings, both in normal and pathological specimens, and use the nomenclature of tumours according to the current WHO classification [72], and are recommended for further reading.

### Pre-analytical and methodological considerations

To ensure diagnostic accuracy, particularly on the first work-up, a minimum of 5 mL—preferably 10–15 mL—should be collected [128]. At least 2–3 mL are required for cytology alone, and higher volumes are essential when malignancy is suspected, as sensitivity increases with sample size [97]. From a pre-analytical perspective, the collection of larger CSF volumes up to 30 mL [78] is considered safe, with contemporary data demonstrating that post-lumbar puncture headache is mainly determined by needle characteristics (tip type, gauge) rather than by the amount of CSF removed [4, 67, 78].

Because of its low protein content and weak buffering capacity, CSF is highly susceptible to pre-analytical variations. Samples should be collected in sterile polypropylene tubes. Glass tubes may cause adherence of cells to the tube wall, leading to underestimation of cell counts. Similarly, regular EDTA or sodium fluoride tubes are unsuitable for cytological analysis due to potential cellular degradation and chemical interference [22]. However, the data available on the extent of changes in relation to CSF findings remain limited. For diagnostic purposes, CSF is typically collected sequentially into multiple tubes, allowing assessment of blood contamination in cases of traumatic lumbar puncture and enabling distribution of samples for routine and specialised analyses, including shipping to external laboratories, without the need for subsequent transfer between containers.

Time to processing is critical. After sampling, CSF pH rises from about 7.3 [112] to alkaline values (up to pH 10 in experimental settings) within a few hours [20], promoting cellular autolysis, and these detrimental processes are accelerated by increased ambient temperatures. Exposure to high ambient temperatures should therefore be avoided during transport. Particularly on hot days, temperature-controlled transport may help preserve cytomorphological integrity [87], although available data on routine refrigeration of CSF are heterogeneous and rapid processing remains the primary pre-analytical priority. Without stabilization, leukocyte loss can be rapid: processing CSF samples within 1–2 h is therefore considered critical. Delays may result in significant reduction of lymphocytes ranging from − 10 to − 53% within 5 h and up to − 60% within 24 h as reported for lumbar and ventricular CSF by studies in neonatal and adult patients [17, 29, 77, 99, 121]. For longer periods, a study by Schwenkenbecher et al. showed that after 72 h 40 to 78% of cells were lytic in samples with blood contamination [115]. This can result in misdiagnosis, particularly in samples with mild pleocytosis.

Neutrophils and tumour cells are considered even more vulnerable. If full cytological workup is delayed, at least a prompt automated or manual cell count should be performed within 2 h followed by slide preparation as soon as possible. There is limited data on the degree of autolysis over longer time periods. These analytical and pre-analytical factors must be carefully considered when interpreting cell counts.

#### Automated vs. manual cell counting

Counting chambers, such as the widely used Fuchs-Rosenthal chamber, are commonly employed for manual CSF cell counting. Freshly collected native CSF (collected less than two hours prior to examination) is pipetted into the chamber (total volume 3.2 µL). As sedimentation can occur even during short transport times, the tube should be gently swirled several times before pipetting. After pipetting, sedimentation should be allowed to proceed for a few minutes. Both anuclear erythrocytes, which can usually be easily distinguished even in an unstained preparation, and remaining nucleated cells are counted individually. As it is usually not possible to distinguish reliably between leukocytes and other nucleated cells (e.g., bone marrow cells) in an unstained preparation, it should be noted that a “false” increase in cell count may be reported, which does not reflect genuine CNS pathology. This should be noted by the cytologist when evaluating the subsequent cytological findings and represents a key argument for the diagnostic relevance of CSF cytology, as microscopic evaluation is required to identify the nature and origin of nucleated cells and to avoid misinterpretation of quantitative results.

In the case of cell-rich or blood-contaminated CSF, dilution with 0.9% NaCl (e.g., 1:10) can be performed for cell counting. The dilution factor is then considered in the cell-count output, as the output unit is always given as “cells per microliter” for reasons of comprehensibility and comparability.

A growing number of laboratories implement automated haematology analysers for CSF cell counting. Automation improves turnaround time and standardization and can perform well at higher cell counts, and in heavily haemorrhagic neurosurgical samples with high erythrocyte and nucleated cell counts, where acceptable precision and clear workflow advantages have been reported [136].

However, despite technical improvements, a reliable lower limit of quantification for CSF leukocytes is still around 20 cells/µL (device dependent) and older systems may have limits around 50 cells/µL [134, 136, 143], if device-specific reference limits are not adjusted and validated. Consequently, expert appraisal concludes that cell counts below 30 cells/µL require mandatory manual confirmation by chamber counting. The systematic review and meta-analysis by Waldrop et al. [134] again highlighted that data on the accuracy of automated counting in the very low range near the reference interval are sparse, and subgroup analyses show markedly weaker correlations for counts <20 cells/µL, although it found good overall agreement between automated analysers and manual microscopy across studies, with stronger agreement at higher absolute counts. Our targeted update identified 15 additional comparative studies covering established haematology analysers as well as newer image-based and semi-automated platforms Supplementary Table S1 (Additional file 1). Overall, these studies reported good agreement with manual chamber counting for total leukocyte counts, particularly at higher cell concentrations. Although some newer platforms showed encouraging performance at low cell counts, the available evidence remained device-dependent and was frequently based on small, single-centre cohorts. Clinically relevant discrepancies around the 5 cells/µL decision threshold were still observed. Automated differential counts generally showed weaker agreement, and atypical or malignant cells could not be reliably identified without morphological review. Thus, the updated evidence broadens the range of evaluated platforms but does not materially alter the conclusions of Waldrop et al. [134].

In summary, a balanced recommendation emerges: manual chamber counting by trained personnel remains the gold standard for quantitative CSF leukocyte assessment, while automated analysers may be used as a first-line approach to ensure availability and rapid turnaround, provided that low and slightly elevated cell counts are confirmed manually and methodological limitations are acknowledged.

A quantitative leukocyte count should be obtained whenever native CSF is available and should not be replaced by an approximate estimate of cells per high-power field on cytospin preparations [128]. Cell recovery on cytospin slides depends substantially on the preparation technique, centrifugation conditions, sample volume, and deposition area, while the area represented by a high-power field additionally varies between microscope systems [63, 104]. Accordingly, universal numerical thresholds based on cells per high-power field are not standardized and should not be used to classify CSF as normocellular or pleocytic [63, 104]. Only in exceptional circumstances in which no native CSF is available for quantitative counting, for example when reviewing an externally prepared slide, may cellularity be described qualitatively, with explicit indication that no quantitative cell count was available.

Apart from automated cell counting, automated cell differentiation provided by routine analysers is—at best—coarse (e.g., granulocytes vs lymphocytes vs monocytes) and should be considered for screening only. Critically, automated systems will not reliably recognize cytomorphological entities that drive CSF differential diagnosis (e.g., plasma cells, activated/reactive lymphocytes, blasts, siderophages/erythrophages, scattered tumour cells, or cell clusters) [113, 136, 142]. Therefore, microscopy remains required whenever qualitative interpretation matters.

Normal adult lumbar CSF leukocyte count reference ranges were not defined in a single landmark trial, but emerged historically from multiple descriptive series of “normal” patients and were later consolidated in laboratory and textbook reference ranges [2, 7]. These ranges appear to be essentially population‑based statistical reference intervals derived from cohorts without clinical or laboratory evidence of CNS disease, then rounded to pragmatic cutoffs for routine.

#### Blood correction in CSF cell counts

Blood admixture is one of the most frequent challenges in CSF interpretation. It can occur during lumbar puncture and may significantly affect cytological and biochemical parameters. As an approximation, about 1 leukocyte is introduced per 1000 erythrocytes. Correction formulas for white blood cell (WBC) count determined by linear regression for samples with strong blood admixture (more than 100,000 red blood cells (RBCs) per µL) seem to be inaccurate and tend to overestimate the number of leukocytes added, potentially leading to underestimation of the true WBC count [56]. Moreover, in patients with significant blood-count changes—e.g., in the context of leukaemia, active systemic infections, anaemia or leukopenia—the ratio may be significantly shifted, leading to inaccuracy in the aforementioned correction factor. In such cases, we recommend considering the individual ratio between erythrocytes and leukocytes in the actual patient's blood count.

To minimize blood contamination, CSF cell count should be performed from the last tube, and serial tubes should be inspected for clearance of blood contamination.

### Cell differentiation

Since CSF is usually a nearly acellular fluid, cells should be concentrated onto slides to enhance diagnostic accuracy. Today, cyto-centrifugation methods (cytospin) are primarily used for this purpose in accordance with current standards [124]. After air drying the cytocentrifuge slide, staining can be performed. The Pappenheim (also known as May-Grünwald-Giemsa) stain has become the standard cytological staining method in CSF laboratories [22, 128]. Other staining methods are available for specific investigations (e.g., Gram, Prussian blue, India Ink, Ziehl–Neelsen), but are used less often given the availability of refined molecular biology and imaging techniques. Despite its subjective nature, expert microscopic examination of stained cytocentrifuge preparation remains the reference standard for qualitative cell differentiation [128]. Trained cytologists can detect subtle morphological clues such as reactive lymphocytes, plasma cells, activated macrophages, granulocytes, atypical and malignant cells, mitotic figures, as well as bacteria—findings critical in neoplastic or inflammatory disorders. Moreover, cytological examination enables the distinction between genuine pleocytosis and falsely elevated cell counts caused by non-leukocytic or artefactual cells that cannot be reliably identified by automated methods or unstained preparations.

## Normal CSF cytology

**Fig. 1:**
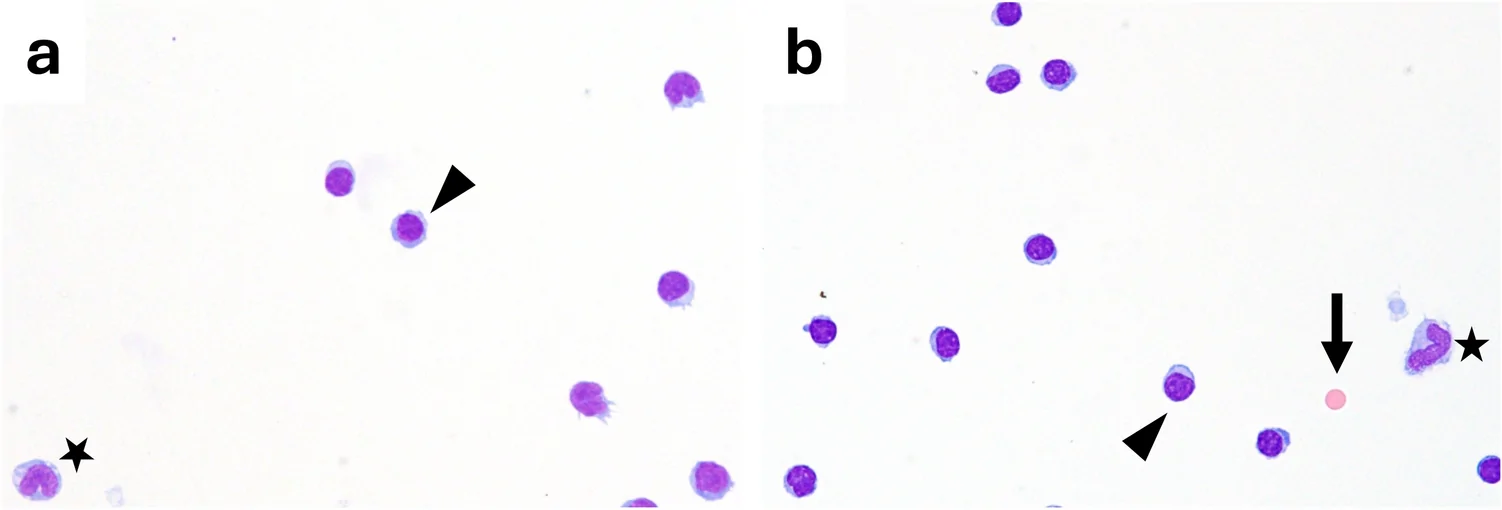
Normal cerebrospinal fluid cytology (Pappenheim stain). Representative cytospin preparations of cerebrospinal fluid stained with Pappenheim stain, showing low cellularity with predominance of small mature lymphocytes (arrowheads) and occasional monocytes (asterisks). Lymphocytes display a round, densely stained nucleus with scant cytoplasm, whereas monocytes are larger with more abundant, lightly basophilic cytoplasm and less condensed chromatin. In (**b**), a single erythrocyte (arrow) is visible, consistent with minimal blood contamination without diagnostic relevance. Overall, no cytological signs of inflammation or malignancy are present (Alt text: representative CSF cytospin showing predominantly small mature lymphocytes and occasional monocytes without pathological abnormalities.)

Cells detected in CSF under physiological conditions are almost exclusively immune cells and do not originate from neural tissue. The blood-CSF and blood–brain barriers effectively prevent the passive passage of erythrocytes and granulocytes, while allowing limited, tightly regulated migration of lymphocytes and monocytes for immune surveillance [31, 98]. This concept underlies the diagnostic value of CSF cell counts, as quantitative and qualitative deviations provide sensitive indicators of inflammatory processes within the central nervous system. Normal adult CSF (Fig. 1) contains ≤ 4 cells/µL, composed predominantly of lymphocytes and monocytes in a roughly 70:30 ratio.

It should be noted that differential cell counts in manual routine CSF cytology are commonly based on the classification of 100 randomly identified cells. Consequently, the resulting percentages represent point estimates subject to binomial variation. For example, a measured proportion of 30% corresponds to an approximate 95% confidence interval of 21–40%, and similar margins apply to other proportions [107]. Minor deviations from the expected distribution may therefore reflect sampling variability rather than true biological differences.

On Pappenheim stained cytospins, nuclei show purple-violet basophilia and cytoplasm appears pale to grey-blue, which allows reliable distinction of leukocyte classes. Lymphocytes are small (≈ 7–9 µm), round cells with dense, homogeneous chromatin and a scant rim of basophilic cytoplasm. Activated lymphocytes are larger than quiescent lymphocytes and uncommon in normal CSF. Importantly, the distinction between resting and activated lymphocytes requires cautious interpretation, as minor variations in cell size or cytoplasmic volume may occur even in non-pathological samples. Premature classification of lymphocytes as “activated” is therefore a common source of over-interpretation in routine CSF differential cytology. Monocytes are about twice the size (≈ 15–20 µm) compared to lymphocytes and morphologically more heterogeneous in non-activated states, typically with indented, kidney-shaped nuclei, fine chromatin, and grey-blue cytoplasm that may contain small vacuoles even without overt activation.

### Artefacts

**Fig. 2:**
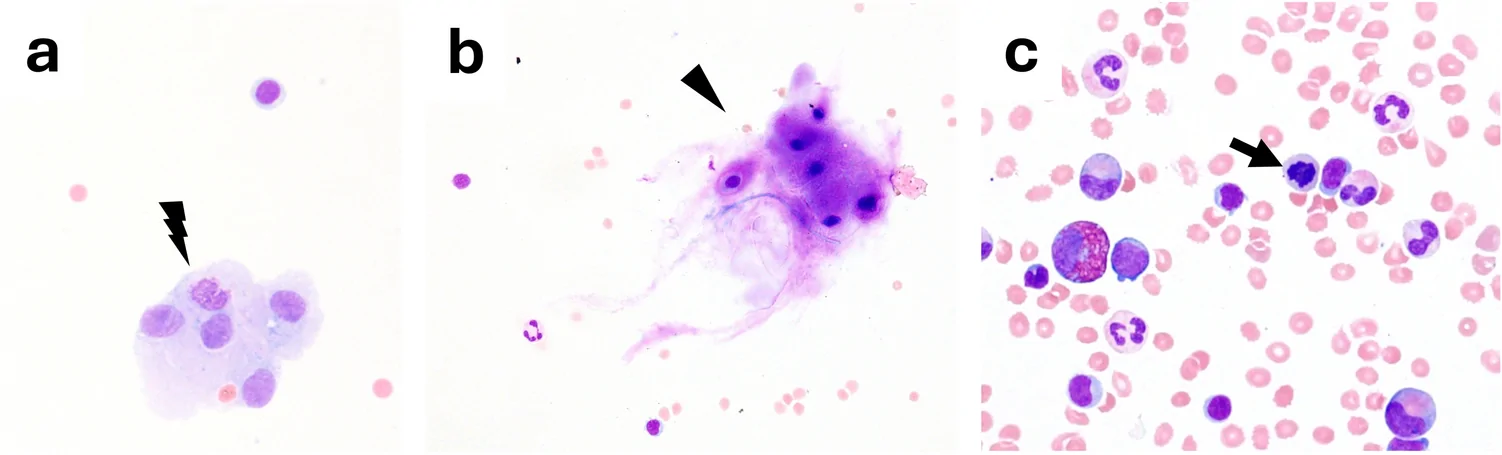
Cytological artefacts in cerebrospinal fluid (Pappenheim stain). Representative cytospin preparations illustrating common cytological artefacts encountered in cerebrospinal fluid analysis. **a** Endothelial cells (lightning bolt), characterized by cohesive clusters of large polygonal cells with abundant pale cytoplasm and oval nuclei, typically introduced by the puncture procedure; **b** cartilage cells (arrowhead), showing large cells with abundant basophilic cytoplasm and round nuclei, representing contamination from intervertebral disc material during puncture; **c** bone marrow contamination, characterized by the presence of haematopoietic precursor cells (normoblast; arrow) of varying maturation stages within a background of erythrocytes (Alt text: representative cytological artefacts in CSF including endothelial cells, cartilage contamination and bone marrow admixture.)

Artefacts (Fig. 2), i.e., cells and phenomena seen on the slide that do not represent the situation in the patient’s CSF in vivo*,* may arise in two distinct ways: firstly, cells or particles may enter the sample during the puncture procedure. Second, artefacts may be added, or emerge, during the preparation and staining procedure.

Erythrocytes are absent unless introduced during the tap procedure (artificially) or via a preceding (subarachnoid) haemorrhage. Artificial blood contamination of varying intensity has been detected in up to 70% of all CSF samples taken, with the incidence appearing to be even higher in infants and neonates [116].

During lumbar puncture, cell types from traversed tissues (e.g., skin, muscle, cartilage, bone marrow, epithelial cells) may appear as artefacts in addition to the otherwise unremarkable differential cytology. Endothelial cells (Fig. 2a) may appear as cohesive clusters of large polygonal cells with abundant pale cytoplasm and oval nuclei. Cartilage (Fig. 2b) presents as chondrocytes in lacunae embedded in a basophilic, chondroid matrix (often metachromatic). CSF samples from the ventricular system may also contain plexus epithelial cells, ependymal cells, and fragments of (degenerating) brain parenchyma. Ependymal or choroid-plexus cells form cohesive sheets or honeycomb clusters of cuboidal cells, sometimes ciliated with uniform round nuclei and delicate chromatin: these should be recognized as site-related contaminants, not carcinoma. Skeletal muscle from the puncture tract appears as elongated fibres or polygonal fragments with dense, fibrillar cytoplasm and peripheral nuclei (striations may be faint on Pappenheim). Smooth muscle cells show spindle cells with “cigar-shaped” nuclei. Bone-marrow carry-over (rare) produces a trilineage mix (Fig. 2c): erythroblasts, myelocytes, and occasional megakaryocytic fragments. Notably, bone marrow admixture will be accompanied by the presence of erythrocytes.

As stated before, contamination can lead to artificially increased cell counts depending on the counting method applied, and must be documented in the report, with cytology used to confirm their nature.

Finally, artificial bacterial admixture (skin flora or reagent precipitates) may mimic infection. The presence of bacteria usually found in “empty voids” between cells, and occurrence without pleocytosis or neutrophils is suspicious for artificial contamination and should prompt clinical correlation.

**Fig. 3:**
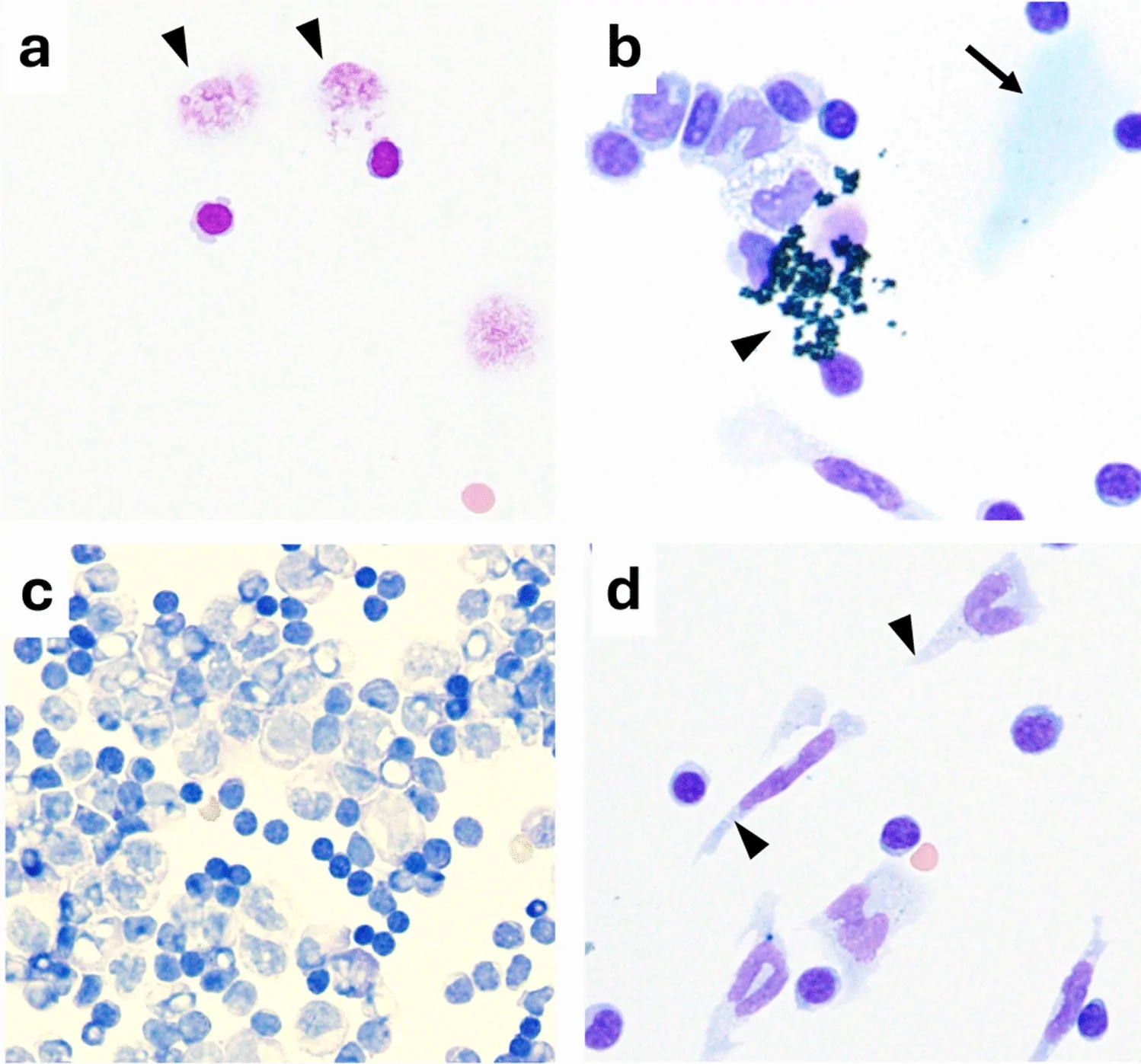
Representative preparation and staining artefacts in cerebrospinal fluid cytology (Pappenheim stain). **a** Autolytic changes with shadow cells (arrowheads), characterized by loss of cellular and nuclear detail. **b** Staining artefacts with granular basophilic material compatible with stain precipitate (arrowhead) and localized uneven background staining/stain pooling (arrow). **c** Overloaded, cell-rich cytospin preparation showing uneven staining with reduced nuclear and cytoplasmic contrast. **d** Cytospin-related mechanical distortion with elongated and altered cellular morphology (arrowheads) (Alt text: representative preparation and staining artefacts in CSF cytology, including cytospin-related cell distortion, an overloaded unevenly stained preparation, autolytic shadow cells, and granular/basophilic staining artefacts.)

Apart from artificial admixture of cells or microorganisms, cell-loss and staining artefacts can impact diagnostic accuracy. Because CSF is protein-poor and weakly buffered, autolysis begins within hours and first affects granulocytes [88]. It has been hypothesized that tumour cells undergo an even more rapid degradation [41]. Typical signs of autolysis include karyolysis or karyorrhexis, smudged nuclei, loss of cytoplasmic granularity, vacuolation, and “shadow” cells (Fig. 3a). Autolysis can artificially lower apparent cell counts and blur differential diagnoses. Advanced autolysis can significantly reduce the diagnostic accuracy of a differential cell count, and therefore should be annotated by the cytologist.

The most common staining artefact in Pappenheim staining includes homogeneously deep-purple granules of variable size that sit in a different focal plane from cells and can mimic bacteria (Fig. 3b). Those granules usually occur when reagents are old or rinsing is inadequate. Furthermore, accurate staining requires phosphate buffer at ~ pH 6.8. pH drift yields muddy cytoplasm and poor nuclear detail (under-stained). Cell-rich samples have to be diluted before staining, because overloaded cytospins produce a multilayer instead of a monolayer: dyes and buffer penetrate unevenly, so the centre appears under-stained (pale nuclei, washed-out cytoplasm), while edges look over-stained with a muddy background (Fig. 3c). Another typical preparation artefact is produced by cytospin preparation. On cytospins, high g-forces can distort nuclei in fragile cells and exaggerate size or alter cell shape (Fig. 3d).

## Cytological patterns in inflammatory CNS disorders

In the CSF, inflammation is reflected by shifts in the differential cell count and by activation of immune cell lines. Understanding activation patterns can provide key insights into the underlying pathology. However, inflammatory cytological patterns are not disease-specific and should not be interpreted as providing aetiological assignment on their own. Their appearance may be influenced by disease phase, antimicrobial or immunosuppressive treatment, intrathecal therapy, blood contamination, and the site of CSF sampling, and should therefore always be interpreted in the clinical and laboratory context.

***Reactive lymphocytes*** enlarge (≈ 10–20 µm), with open chromatin and more abundant basophilic cytoplasm, and variable nuclear contours. Nuclear indentations, irregular nuclear outlines, and occasional nucleoli may occur as part of reactive activation and should not per se be interpreted as malignant features. However, pronounced cytoplasmic irregularity and deep nuclear notches have been reported significantly more frequently in neoplastic than in inflammatory lymphocytes [92]. Importantly, the majority of activated lymphocytes observed in inflammatory CSF conditions represent activated T-lymphocytes, rather than B-cell lineage differentiation [49]. In highly activated CSF (e.g., acute neuroborreliosis), mitoses of lymphocytes can occasionally be detected. ***Plasma cells*** represent terminally differentiated B-cells [94] and therefore reflect advanced humoral immune activation rather than a mere activation state. Cytologically, they are characterized by an eccentrically flattened nucleus with coarse chromatin, a perinuclear pale zone corresponding to the Golgi apparatus, and deeply basophilic cytoplasm. The presence of plasma cells in CSF may precede the quantitative detection of intrathecal immunoglobulin synthesis, underscoring the added diagnostic value of cytological examination in inflammatory CNS disorders.

***Monocytes*** gradually transform to activated macrophages with enlargement and vacuolation of the cytoplasm. Phagocytosis of cellular debris is common in viral or resolving bacterial meningitis. Activated monocytes can become remarkably enlarged due to numerous vacuoles, and the cytoplasm can appear foamy (“foam cells”) or—in the case of individual, large vacuoles—almost empty (“signet ring cell”).

***Neutrophil granulocytes*** appear round and have segmented nuclei. During acute phases, such as fulminant bacterial inflammation, immature neutrophils with “band-shaped” nuclei may be observed. During inflammation, an increase in hyper-segmentation to usually 4–6 nuclear segments, occurs. It has been reported that scattered neutrophils, which can nevertheless occur in “normal” CSF without pleocytosis, are not associated with CSF infection [13, 24], although there is little evidence for this assumption in the literature.

***Eosinophil granulocytes*** usually have distinct eosinophilic granulated cytoplasm and, in most cases, two clearly recognizable nuclear segments. Eosinophil-dominated pleocytosis is rare and usually indicates the presence of a parasitic or coccidioidal CNS infection, or, more rarely, a concomitant reaction in neoplastic meningitis, especially reported in CNS involvement of Hodgkin’s disease, or a shunt-related reaction [36, 43, 46]. In certain autoimmune inflammatory CNS disorders, most notably AQP4-IgG-positive neuromyelitis optica spectrum disorder (NMOSD), a mixed pleocytosis with a substantial proportion of eosinophilic granulocytes has been reported [50]. Rarely, CSF eosinophilia has also been reported in vasculitic CNS disorders, including eosinophilic granulomatosis with polyangiitis and primary angiitis of the central nervous system [45, 86]. CSF eosinophilia may also occur in drug-induced aseptic meningitis, including cases associated with NSAIDs and antibiotics [80].

***Basophilic granulocytes*** are distinctly deep blue or basophilic granulated in the cytoplasm, making it difficult to clearly distinguish between the nucleus and plasma in everyday practice. They are rarely present in CSF, and their diagnostic significance remains poorly understood. In non-blood-contaminated and otherwise unremarkable CSF, basophils are not expected. When identified, their presence should therefore be interpreted in the context of blood contamination, haematological or leptomeningeal neoplastic involvement, and, exceptionally, inflammatory CNS disorders such as neurosarcoidosis or viral meningitis [18, 19, 25].

### Infectious CNS inflammation

#### Bacterial

**Fig. 4:**
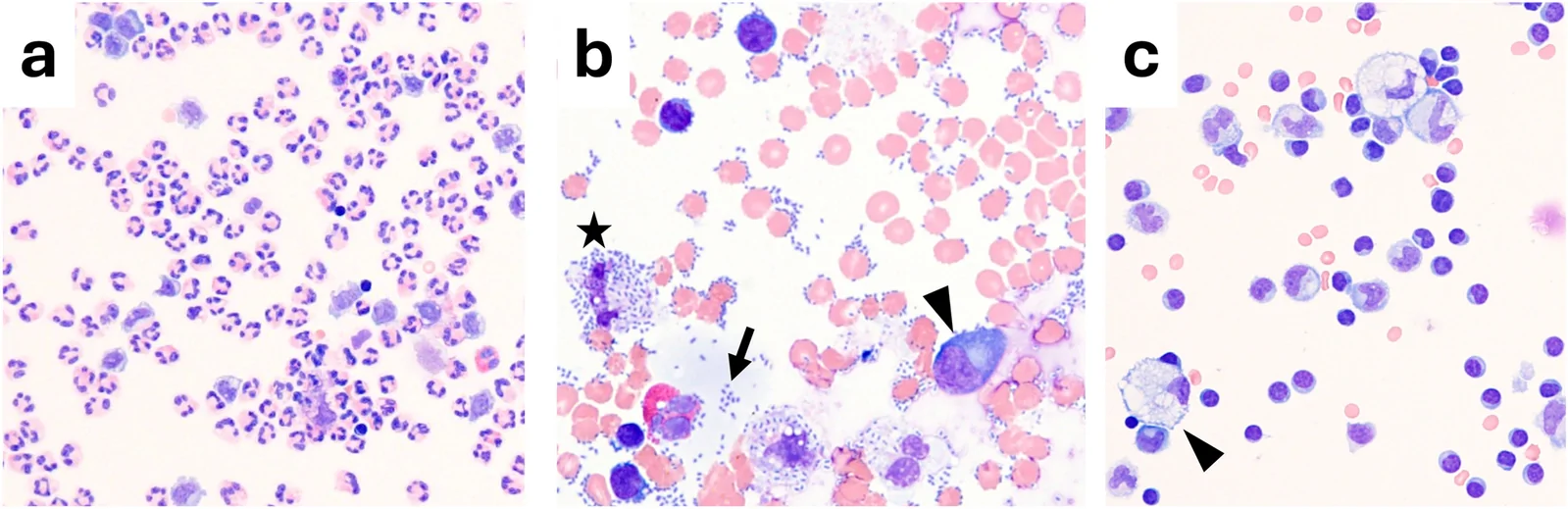
Cytological patterns of infectious inflammatory CNS disease (Pappenheim stain). Representative cytospin preparations of cerebrospinal fluid illustrating different infection-associated inflammatory patterns. **a** Pronounced neutrophilic pleocytosis in bacterial (purulent) meningitis without detectable microorganisms. **b** Early apurulent meningoencephalitis in Streptococcus pneumoniae infection, characterized by the presence of extra- (arrow) and intracellular (asterisks) diplococci in the absence of a predominant neutrophilic infiltrate, accompanied by a large plasma cell (arrowhead). **c** Later stage of viral CNS inflammation with resorptive changes, characterized by macrophages with foamy cytoplasm (“foam cells”, arrowhead) reflecting phagocytic clearance of cellular debris (Alt text: three CSF cytospin micrographs showing (**a**) marked neutrophilic pleocytosis, (**b**) intra- and extracellular diplococci with a plasma cell despite limited neutrophilic inflammation, and (**c**) foamy macrophages during resolving inflammation)

Acute purulent bacterial meningitis is a neurological emergency with substantial case fatality and long-term morbidity among survivors [5]. Regarding the cytological CSF findings, high cell counts (usually exceeding several 1000 cells per µL) with neutrophil predominance (Fig. 4a) are typically found. In adults, neutrophils typically constitute > 90% of leukocytes in classic presentations [128, 129].

Nevertheless, it should be emphasized, that infections naturally progress in phases, with different findings expected in the early or late stages of bacterial CNS infections. In the early phase of bacterial meningitis (approximately 12 h or less) [32]—as particularly described in cases of meningococcal and pneumococcal disease—bacteria may be directly detectable in the specimen alongside a lower inflammatory response and, during this period, the cell count may appear normal or near to normal, with only mild signs of inflammation present, sometimes even without evidence of neutrophilic infiltration (Fig. 4b). This particular case of bacterial meningitis is also known as “apurulent” meningitis and is considered particularly threatening. It occurs more frequently in elderly patients and in the context of immunocompromising conditions [118].

As inflammation progresses, the ongoing immune response leads to degradation processes (Fig. 4c), resulting in a decrease in neutrophilia and a relative increase in macrophages, activated monocytes and lymphocytes. Initiation of antibiotic treatment before lumbar puncture (according to guideline procedures) can sterilize CSF within a few hours, but does not substantially change leukocyte counts within the first hours [55].

Another scenario that can lead to deviations from expected findings is immunosuppression. In severely immunocompromised patients, lower pleocytosis [132] or even normal leukocyte counts [118] may occur more frequently due to a significantly reduced inflammatory host response. Therefore, normal leukocyte count does not rule out infection. In such cases, diagnosis relies more readily on further direct or indirect pathogen diagnostics, as well as the patient history and clinical course.

#### Neuroborreliosis

In neuroborreliosis, usually moderate pleocytosis between 50 and 500 cells per µL is seen [128]. A relevant proportion of neutrophilic granulocytes might be present, especially in early infection and before treatment [89]. Due to the presence of strong lymphocytic activation and often remarkably increased plasma cell frequency and even the presence of mitotic figures, it may be difficult to distinguish from neoplastic meningitis at first glance based on morphology alone. However, in lymphoma slightly to moderately activated (“intermediate”) lymphocytes will usually be absent. In diagnostically ambiguous cases, extended investigations are required. These include flow cytometry, an integrated CSF report incorporating intrathecal immunoglobulin synthesis and blood-CSF barrier function assessed by Reiber diagrams, CXCL-13, Borrelia-specific antibody index calculation, and Borrelia-specific serological testing [103, 127, 138].

#### Viral, tuberculous, and fungal

In theory, a similar phasic course involving initial neutrophil infiltration is also assumed for viral infections [15, 70]. However, the transition to lymphocyte-dominated findings appears to occur more rapidly in these cases than with most bacterial pathogens: interval CSF analyses in bacterial meningitis have shown that pleocytosis and neutrophil predominance frequently persist beyond 48–72 h, even under effective antimicrobial therapy [10, 70]. However, systematic data directly comparing the temporal dynamics of CSF cellular changes across different infectious etiologies remain limited. Viral CNS infections usually show lymphocytic pleocytosis (typically 50–500 cells per µL) by the time of diagnostic lumbar puncture [70]. Nevertheless, for several virus types like enterovirus, West Nile, FSME, or HSV, CSF findings may transiently lack pleocytosis or show a variable neutrophilic skew [15]. Leukocyte counts are usually slightly to moderately elevated. Opportunistic infections of the central nervous system (e.g., Human polyomavirus 2 (formerly JCV), CMV) can cause inconspicuous cytological findings, so they often can only be detected through advanced diagnostics.

While an early neutrophilic response with subsequent rapid lymphocytic predominance is well established in viral CNS infections [70], evidence for a comparable phasic course in tuberculous or fungal infections is limited, and CSF cytology in these conditions often shows heterogeneous or mixed cellular patterns.

Tuberculous meningitis typically presents with a moderately to markedly elevated CSF white blood cell count ranging from approximately 50 to 1000 leukocytes per µL, characterized by a mixed cellular composition with variable proportions of lymphocytes and neutrophilic granulocytes, particularly in early disease stages, and a shift toward lymphocytic predominance with disease progression or under therapy [122]. Occasionally, eosinophils have been reported in tuberculous meningitis and may contribute to a heterogeneous cytological appearance [66]. In CNS tuberculosis, Ziehl–Neelsen staining reveals acid-fast bacilli in only a minority even of otherwise confirmed cases [122]. Thus, nucleic acid amplification has become the gold standard for diagnosis, and larger CSF volumes (7–10 mL) should be drawn routinely to enhance diagnostic sensitivity, particularly in tuberculous meningitis, because Mycobacterium tuberculosis is often present in very low concentrations in CSF [27].

Fungal CNS infections are associated with a highly heterogeneous CSF inflammatory profile that varies with pathogen, disease stage, and host immune status [69]. Similar to tuberculous meningitis, fungal pathogens are often present at low concentrations in CSF, rendering microbiological and molecular diagnostics strongly dependent on adequate sample volume.

### Autoimmune inflammation

CSF cytology in non-infectious autoimmune disorders is typically lympho-monocytic and of low-to-moderate extent, but certain entities show distinctive features. Interpretation should integrate clinical findings and further diagnostic testing. Cytology usually supports but is not sufficient to establish these diagnoses.

Multiple sclerosis is the most common immune CNS disease that requires detailed CSF diagnostics. Routine CSF parameters are often normal or show at most a mild lymphomonocytic pleocytosis, typically below 50 cells/µL [2]. While mild pleocytosis and signs of lymphocytic activation including immunoglobulin-producing plasma cells have been reported more frequently in relapsing disease courses [6], the strength of correlation between CSF cell count and clinical activity remains debated.

In addition to multiple sclerosis, there are additional autoimmune diseases that can cause CSF inflammation. It is noteworthy that CSF leukocyte count and differential cell cytology in autoimmune processes can morphologically mimic conditions, such as infectious diseases, so that the cumulative findings, considering additional diagnostic tests and follow-up observations, are often of great relevance.

In contrast to multiple sclerosis, both aquaporin-4-IgG-positive NMOSD and MOG-antibody-associated disease (MOGAD) display more variable and often more pronounced pleocytosis (up to 500 cells per µL) during attacks, with frequent neutrophils and occasional eosinophils, particularly in NMOSD and in paediatric cohorts—patterns that should raise suspicion when the clinical picture is not compatible with multiple sclerosis—and initially eventually mimic bacterial infection [50, 51].

Autoimmune encephalitis (AIE) does not necessarily present with CSF pleocytosis. Especially in AIE subtypes that occur in elderly patients, CSF leukocyte count is often normal [8]. However, if present, mild to moderate lymphocytic pleocytosis is common, and neutrophils [57] can appear in the very acute phase of some AIEs, which may mimic infectious disease.

### Other causes of CSF inflammation

**Fig. 5:**
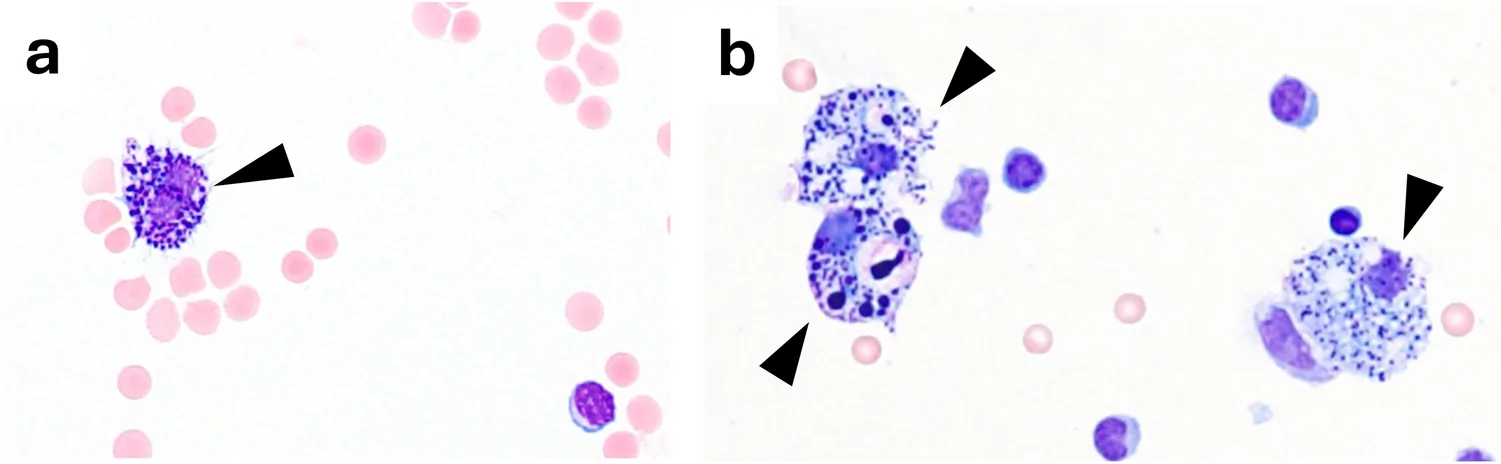
Antisense oligonucleotide-associated macrophage inclusions in cerebrospinal fluid (Pappenheim stain). Representative cytospin preparations of cerebrospinal fluid showing macrophages with numerous sharply condensed, basophilic to purple intracellular inclusions after intrathecal antisense oligonucleotide therapy. **a** Single macrophage with densely packed cytoplasmic inclusions consistent with a nusinersen-associated macrophage inclusion cell (“nusinophage”; arrowhead) in an otherwise paucicellular preparation with mild artificial blood contamination. **b** Tofersen-associated macrophage inclusions (“tofersenophages”; arrowheads) with numerous variably sized basophilic to purple inclusions distributed throughout the cytoplasm (Alt text: two CSF cytospin micrographs showing macrophages with purple-blue cytoplasmic inclusions following intrathecal antisense oligonucleotide therapy: (**a**) nusinersen-associated and (**b**) tofersen-associated inclusions.)

Intrathecal therapies may provoke distinctive cytological changes. During nusinersen treatment in patients with spinal muscular atrophy, macrophages with numerous sharply defined purple/blue inclusions (“nusinophages”; Fig. 5a) appear without sustained inflammatory shifts [40]. Similar macrophage inclusions have been described in patients receiving intrathecal Tofersen (Fig. 5b) [119]. Tofersen for SOD1-mutant amyotrophic lateral sclerosis is associated with frequent elevations of CSF leukocyte count, intrathecal immunoglobulin synthesis and CSF protein, and occasional aseptic/chemical meningitis or myelitis. These laboratory changes are often asymptomatic but have to be recognized as treatment-related [137]. Intrathecal steroids [126] and other agents can also cause sterile neutrophilic pleocytosis (“chemical meningitis”). In addition, intravenous immunoglobulin (IVIG) therapy is a well-recognized cause of aseptic meningitis, typically presenting within hours to days after infusion and characterized by a marked pleocytosis, often neutrophil-predominant in the early phase, with elevated CSF protein but negative microbiological studies [58, 65]. Finally, mild “reactive pleocytosis” can follow repeated lumbar punctures within short intervals [110].

Mild inflammatory CSF changes may occasionally be observed in the absence of infection, autoimmune disease, or recent lumbar puncture. In such cases, tissue damage of the central nervous system, for example following surgery, trauma, or ischemic stroke, can induce reactive inflammatory changes detectable in CSF cytology [100].

## CNS haemorrhage

It is well known that the imaging findings obtained by cranial computed tomography (CT) may be unremarkable in a relevant proportion of patients with subarachnoid haemorrhage (SAH), especially in subacute scenarios [82, 135]. In these cases, visual inspection of CSF colour, persisting xanthochromia of the supernatant after centrifugation, testing chemical parameters such as ferritin, and most importantly, targeted CSF cytopathological analysis provides decisive evidence [82]. Neither xanthochromia nor a remarkably elevated erythrocyte count are specific for SAH. CSF cytology yields time-dependent hallmarks of prior haemorrhage. After bleeding, macrophages that phagocytose blood components can be detected in CSF.

**Fig. 6:**
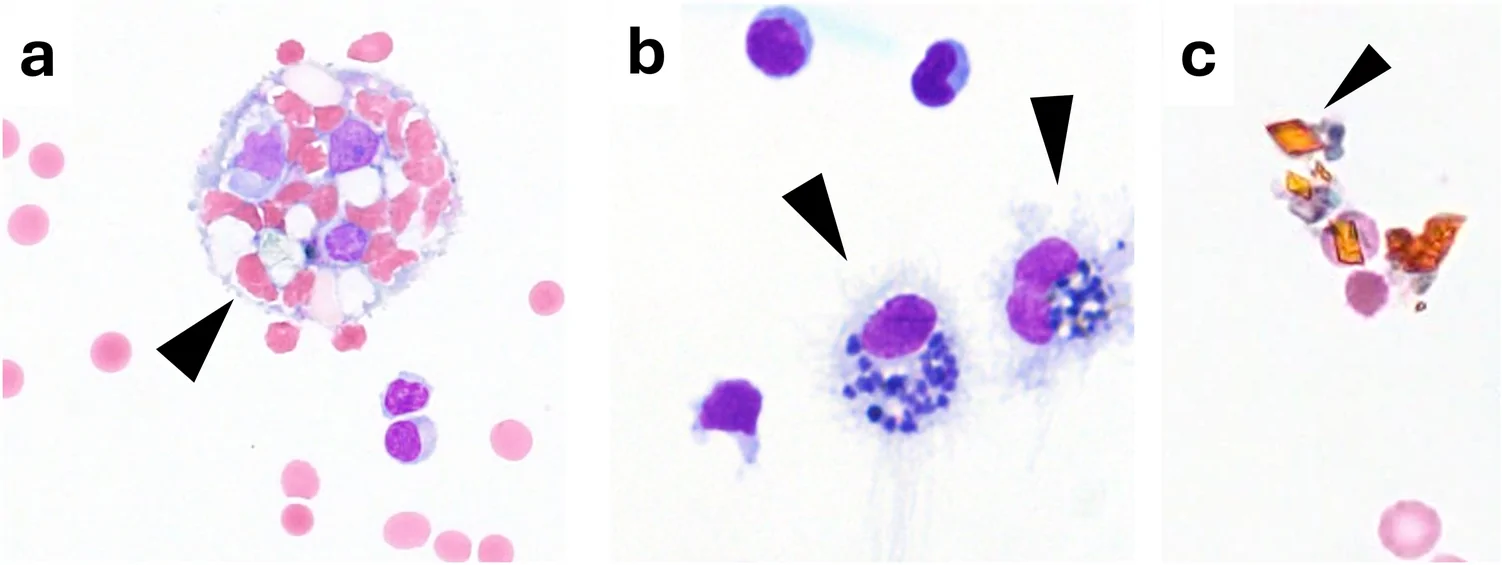
Cytological markers of different stages of haemorrhage in the CSF (Pappenheim stain). **a** Erythro(leuko)phage (arrowhead), indicating recent haemorrhage, containing intact erythrocytes and occasional leukocytic remnants. **b** Siderophages (arrowhead), reflecting subacute haemorrhage, with intracellular iron-containing granules resulting from haemoglobin degradation. **c** Extracellular haematoidin crystals (arrowhead), representing late-stage haemorrhage, appearing as yellow-orange, birefringent crystalline structures within the extracellular space (Alt text: representative cytological findings associated with intracranial haemorrhage including erythrophages, siderophages and haematoidin-crystals.)

The first subtype of macrophages, which can usually be observed within 4–12 h after the event, are erythrophages [82, 133]. Within the next days, erythrophages (Fig. 6a) gradually evolve to siderophages (Fig. 6b), which usually occur after 24–48 h [82, 133]. They contain granular dark pigment (hemosiderin) and stain positively with Prussian Blue (which may be used in cases of doubt). Intermediate mixed erythro-/siderophages (erythro-/haemosiderophages) may occur during haemoglobin degradation and contain both partially preserved erythrocytes and intracellular haemosiderin granules. They represent transitional forms between erythrophages and siderophages and should therefore not be interpreted as a separate stage with a sharply defined time window [61, 62, 124]. In sequential samples, the concomitant presence of fresh erythrophagocytosis and more advanced haemosiderin-containing macrophages may raise suspicion of recurrent bleeding and should be interpreted together with the clinical and imaging findings [61, 62]. During progressive degradation, phagocytosed erythrocytes may lose their staining properties and appear as optically empty intracytoplasmic vacuoles, potentially mimicking foamy macrophages. Such vacuolated macrophages may raise suspicion of preceding erythrocyte phagocytosis but are non-specific and, in isolation, do not constitute reliable cytomorphological evidence of haemorrhage. Identification of preserved erythrocytes, haemosiderin, or haematoidin provides more specific evidence, and Prussian blue staining may help confirm iron-containing pigment in equivocal cases [82, 124].

Later, characteristic yellow-orange rhomboid or star-like crystalline structures can be detected within the siderophages: the haematoidin (bilirubin) crystals (Fig. 6c). These may be detected floating in CSF for months after macrophage apoptosis. By using polarized-light microscopy, the crystalloid structures can also be made clearly visible. The precise morphological classification of phagocytic cells can be used to draw conclusions about the temporal stage or acuity of bleeding. However, when considered alongside the reappearance of erythrophages in sequential CSF samples, they raise concerns for repeated or persistent bleeding and should prompt correlation with clinical course and neuroimaging.

In the context of intrathecal therapies, a rare diagnostic challenge may be foreign-body macrophages: Since they share a foamy, vacuolated appearance with basophilic inclusions (which often appear less blurred) e.g., of drug remnants such as tofersen or nusinersen on Pappenheim stains, they must not be mistaken for siderophages. Careful correlation with patient history and clinical course, and further staining (e.g., Prussian blue) when in doubt, help avoid misclassification.

For cases of prolonged transportation, there was no evidence that artificial blood admixture in CSF can lead to in-vitro occurrence of erythrophages [23, 115, 133].

## Neoplastic diseases in CSF cytology

Despite major advances in neuroimaging and molecular CSF assays, conventional CSF cytology remains a decisive part of the routine work-up whenever leptomeningeal involvement or a neoplastic CNS process is suspected. Nevertheless, there are a few important pitfalls that must be carefully considered alongside the cytological findings of these suspected diagnoses. Neoplastic CNS diseases such as neoplastic meningitis may lead to marked pleocytosis. However, a normal CSF leukocyte count does not exclude CNS neoplasia [11]. Accordingly, guideline groups advise performing CSF cytology when clinical/MRI findings keep pretest probability high [105] and, if needed, repeated cytological analysis. Whether malignant cells are recovered from CSF seems to depend on histological subtype, anatomic access to the CSF space (subarachnoid/ventricular contact), disease burden, and pre-analytics (sample volume, rapid processing) [97]. Parenchymal masses with intact pial or ependymal boundaries, such as meningiomas, will typically yield negative cytology unless atypical/anaplastic histology or overt leptomeningeal or intraventricular seeding creates a conduit to CSF.

**Fig. 7:**
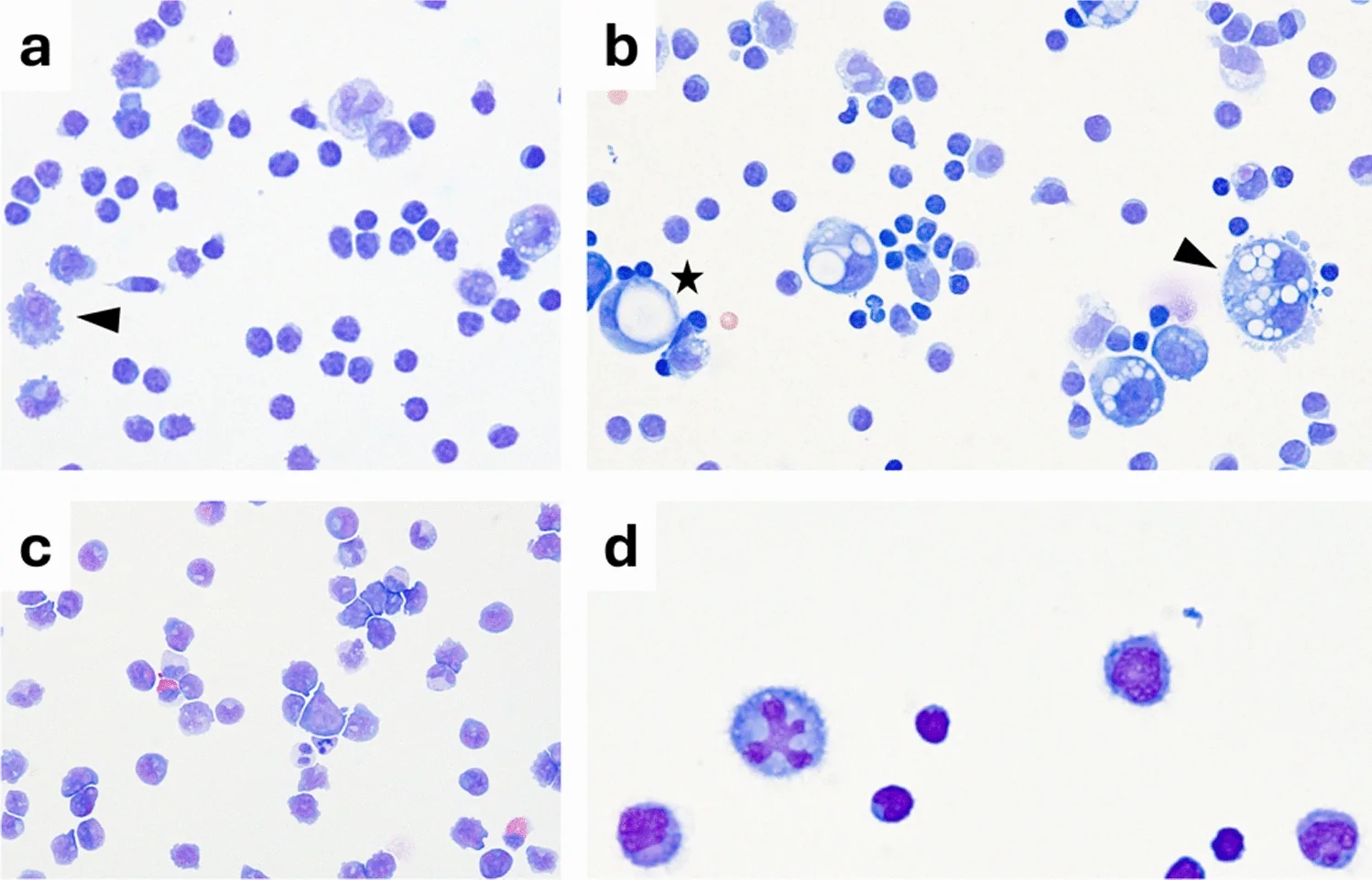
Cytological features of neoplastic CNS involvement (Pappenheim stain). Representative cytospin preparations of cerebrospinal fluid illustrating different forms of neoplastic meningeal and parenchymal involvement. **a** Lymphomatous meningitis (Meningeosis lymphomatosa), showing atypical lymphoid cells with enlarged nuclei, coarse chromatin and a prominent nucleolus (arrowhead). The neoplastic cell additionally displays conspicuous cytoplasmic projections. **b** Carcinomatous meningitis (Meningeosis carcinomatosa), characterized by large malignant epithelial cells with markedly vacuolated cytoplasm, including a signet ring-like cell (asterisk), as well as multinucleated tumour cells with mucin production (arrowhead). **c** Leukemic meningitis (Meningeosis leucaemica), with infiltration by immature haematopoietic cells showing nuclear atypia and variable cell size. **d** Disseminated cerebral germinoma, demonstrating atypical cells with enlarged, irregular nuclei and prominent nuclear abnormalities (Alt text: representative malignant CSF cytology showing neoplastic cells from different haematological and solid tumour entities.)

The appearance of malignant cells (Fig. 7) in the CSF can be highly variable, depending on the underlying neoplastic disease. Experienced examiners base their diagnosis on the morphological criteria typically exhibited by malignant cells [21, 47, 48, 92, 124]:

- Abnormal cell size or shape: enlarged cells, with size varying between single tumour cells (often 20–25 µm in diameter, may be up to 200 µm)
- Abnormal nucleus: enlarged and/or polymorphic, irregular nuclei, visible nucleoli or pseudo-inclusions, nuclear moulding, multinucleation, changes in chromatin structure and distribution
- Atypical mitoses or apoptoses
- Abnormal cytoplasm: e.g., vesicles in adenocarcinoma cells, pseudopod-like extensions from the cytoplasm, a high nucleus-to-plasma ratio, and intensive basophilia of the cytoplasm
- Possibly clustering (in some entities)
- Phagocytosis of tumour cells
- Accompanying inflammatory reaction: lymphocytes (which may be the predominant cell type), monocytes, neutrophils, and erythrocytes

No single cytological parameter is pathognomonic for malignancy in CSF [92]. The higher the number of malignancy criteria found, the more reliable the diagnosis of neoplastic disease becomes. Pre-analytical deterioration may itself create misleading cytomorphological features. In autolytic specimens, progressive nuclear fading may produce an apparent “pseudo-nucleolus”, potentially mimicking a prominent true nucleolus of a malignant cell. In equivocal cases, interpretation should therefore be cautious and reassessment of a promptly processed repeat specimen may be advisable. In melanoma, intra- or extracellular melanin pigment may provide an additional diagnostic clue but should be distinguished from haemoglobin-derived pigment such as haemosiderin. If doubts remain, it may be appropriate to refer to “atypical” cells and diagnose only “suspected neoplastic meningitis”, with the recommendation of repeated puncture and analysis, possibly from a larger CSF volume. Other activated cells (such as lymphocytes, monocytes, and granulocytes) are also frequently found, indicating an accompanying inflammatory reaction, hence the term “neoplastic meningitis”. In cases of tissue damage, erythrocytes may also be present. On the other hand, only few scattered cells with increased size, nuclear atypia, and cytoplasm vesicles may establish the diagnosis of carcinomatous meningitis, e.g. in adenocarcinomas.

When cytology is positive, morphology alone rarely permits definitive tumour classification (exceptions include adenocarcinoma, acute leukaemia, some lymphomas, and melanoma), prompting adjunct techniques like immunocytochemistry, flow cytometry, or biopsy. In haematological malignancies, particularly lymphoid and leukaemic disease, multiparameter flow cytometry may provide higher sensitivity than conventional cytomorphology and should be considered an important complementary method [96, 117]. However, positivity criteria and the clinical significance of flow-cytometry-positive/cytology-negative findings remain incompletely standardised [96]. Selected cell-block preparations may additionally facilitate immunocytochemical or molecular characterisation when sufficient cellular material is available [139]. In leptomeningeal metastasis, newer circulating tumour-cell assays may increase sensitivity compared with conventional cytology, although assay platforms and positivity criteria remain heterogeneous. In addition, CSF liquid biopsy approaches are increasingly being used as complementary diagnostic tools in neuro-oncology. Analysis of circulating tumour cells and cell-free tumour DNA (cfDNA/ctDNA) by targeted sequencing, next-generation sequencing, digital PCR or nanopore-based approaches may increase diagnostic sensitivity and provide information on tumour-specific molecular alterations, particularly in leptomeningeal disease [1, 75, 83]. Beyond mutation and copy-number analysis, methylation profiling of CSF-derived cfDNA is emerging as a promising approach for molecular classification of CNS tumours [1]. These techniques should currently be regarded as complementary to, rather than replacements for, conventional cytology and should be interpreted together with cytomorphology, imaging and the clinical context. For potential downstream molecular analyses, particularly in patients with cancer of unknown primary or multiple possible primary tumours, retention of cell-free CSF supernatant may be considered. Some laboratories use the same CSF specimen for both cytological and molecular analyses by removing and storing the cell-free supernatant after an initial centrifugation step and replacing the removed volume with saline or phosphate-buffered saline before cytospin preparation. However, the impact of such combined workflows on cellular recovery, cytomorphological preservation and diagnostic performance has not yet been systematically validated. The following is recommended for everyday clinical practice: Obtain large CSF volumes, process promptly, and repeat the lumbar puncture if the first specimen is negative but suspicion persists. Across cancers, the first tap’s sensitivity (~ 50–60%) rises to ~ 80% with repeat collections [42].

## CSF cytology in infants and children: age-specific CSF leukocyte counts and differential patterns

### Age-dependent reference ranges: what is “normal” by age?

What is considered a normal CSF cytology finding in an adult cannot be directly applied to CSF diagnostics in early life. In neonates, robust cohort and meta-analytic data indicate low but slightly higher upper reference limits for CSF leukocyte counts compared with older infants and children. In a cohort of 318 infants, the 95th percentile for CSF leukocytes was 12 cells/µL in preterm and 14 cells/µL in term neonates [120]. Although there are few large-scale studies on cell counts in preterm and term CSF, the available data suggests higher cell counts in the neonatal period [144]. Beyond the neonatal period, values approach the ≤ 4 cells/µL range within the first three months of life [59, 81]. The possibility that compression or mechanical stress on the head during labour and birth could cause a higher CSF leukocyte count in neonates was discussed, but could not be confirmed [131]. Further hypotheses include an immature blood-CSF barrier or immature CSF circulation, which may allow immune cells more time to migrate into the CSF due to markedly reduced flow dynamics.

However, there appear to be no reliable clinical or experimental studies. In healthy neonates, CSF differential cell count is predominantly mononuclear, consisting of lymphocytes and monocytes, but a minor neutrophil component can be encountered in otherwise normal CSF. Particularly in preterms, higher proportions of neutrophils appear to occur more frequently [79, 144]. After about 2 months of age, the CSF cell proportions should mirror adult references.

### Diagnostic pitfalls specific for early life

(i) Apurulent meningitis: As is the case in immunosuppressed adult patients, cases of infectious meningitis or encephalitis without evidence of pleocytosis appear to be more prevalent in neonates and young infants than in healthy, immunocompetent adults [145]. In a cohort of 40 neonates and young infants (< 90 days of age) within 44 episodes of PCR- or culture-proven bacterial meningitis, 13 out of 44 episodes (30%) showed CSF leukocyte counts within the normal range [14]. A larger cohort of neonates with culture-proven meningitis reported 6.5% (12/184) of cases with no significant leukocyte count elevation [39]. In contrast, only around 2% of cases of bacterial meningitis in adults present as apurulent [118].

(ii) Blood contamination and correction formulas: Traumatic taps are common in neonates [116]. However, applying a fixed 1000:1 RBC:WBC correction can misclassify cases and mask pleocytosis in bacterial meningitis. In infants ≤ 60 days, a retrospective analysis showed that using a 1000:1 ratio for correction would have reclassified some bacterial meningitis cases as “no pleocytosis” [34]. Two large cohort studies indicated that the application of other correction calculations appeared to be more accurate than 1:1000: In 432 traumatic taps in neonates, a correction ratio of 1:400 was suggested [38], while a cohort of 2880 neonates and young infants < 60 days achieved the most accurate correction with 1:877 [73]. Therefore, for paediatric CSF in general, most authors advise reporting the uncorrected CSF leukocyte count alongside the degree of RBC contamination and interpreting in a clinical context rather than “correcting away” cells.

## Topography of CSF cytology: interpreting ventricular CSF

### Differential cell counts in ventricular CSF

Ventricular CSF (vCSF) is usually sampled in a specific clinical context, typically via an external ventricular drain (EVD) following haemorrhage, neurosurgery, trauma, or hydrocephalus. Tissue particles and the concern for interfering ventriculitis necessitate careful microscopy.

Because cells and larger particles equilibrate unevenly across the CSF spaces, ventricular samples potentially under-represent cellular inflammation compared to lumbar CSF, so a “normal” vCSF leukocyte count does not necessarily mirror distal compartments. Parallel samplings suggest that lumbar leukocyte counts are usually higher than ventricular counts in the same patient, even when infection is present. Most likely this reflects compartmentalization, rostrocaudal gradients, and sedimentation of cellular elements within the CSF space. In comparative series of meningitis cases, lumbar leukocyte count was consistently higher than vCSF leukocyte count within the same illness episode [26, 54, 95]. Although previous findings underscore that site matters for interpretation, there is no consensus on specifying normal ranges in vCSF that deviate from lumbar CSF.

### Diagnosing ventriculitis from a cytologist’s perspective

Although pleocytosis is usually expected in ventriculitis, there appears to be a relevant number of patients without elevated CSF leukocyte count. A comprehensive meta-analysis described increased leukocyte count for 93% of patients with bacterial ventriculitis and, on the other hand, 32% of patients without ventriculitis [28]. Therefore, in healthcare-associated ventriculitis, routine indices are imperfect: normal CSF leukocyte count, glucose, or protein do not reliably exclude infection, particularly after antibiotics or with low-burden or compartmental disease. Guidelines therefore prioritize CSF culture (and repeat testing when suspicion remains) over any single leukocyte count threshold [130]. In practice, vCSF pleocytosis—when present- is usually neutrophil-predominant but can be blunted by prior surgery, intraventricular haemorrhage, or intrathecal therapy, all of which also raise RBC counts and confound interpretation [130].

### Accounting for blood contamination in ventricular CSF

In cases where vCSF is obtained, the presence of blood contamination will frequently be observed. This phenomenon may be attributed to various factors, including recent EVD implantation, postoperative complications, or underlying conditions such as SAH, as well as complications such as ventriculitis. For fixed correction formulas like 1:666 or 1:1000, high percentages of over- or under correction have been reported [9]. Some authors have suggested that blood-corrected CSF leukocyte counts should not be performed in general and therefore uncorrected leukocyte counts should be reported for vCSF. With regard to the diagnosis of assumed CNS-infection (e.g. ventriculitis), a more sophisticated, yet potentially more precise approach involves the implementation of a patient-specific correction index (CI):

$$\mathrm{C}\mathrm{I}= \frac{\mathrm{C}\mathrm{S}\mathrm{F} \mathrm{W}\mathrm{B}\mathrm{C}\div \mathrm{C}\mathrm{S}\mathrm{F} \mathrm{R}\mathrm{B}\mathrm{C}}{\begin{array}{c}blood WBC\div blood RBC\\ \end{array}}.$$

A CI elevation (≥ 4–5-fold) above the blood ratio has been associated with ventriculitis and improves discrimination from “bloody but sterile” CSF in an observational cohort [93]. A recent 2024 multicentre analysis supports CI as a useful adjunct, while noting that cut-offs vary and do not replace culture [37]. In addition, sequential analyses (daily or every other day) will allow for timely detection of an abrupt increase in both CSF leukocyte count and, subsequently, CI in vCSF.

### Device-related eosinophilia and atypical differentials

Eosinophils in vCSF are well-described in shunted or drained patients and correlate with hardware malfunction, CSF extravasation, and sometimes infection (e.g., Cutibacterium acnes) [36]. Eosinophilia can be striking yet non-specific; it should prompt evaluation for mechanical problems and targeted microbiology rather than be dismissed as “allergic” or “reactive”. Neutrophil-rich but culture-negative pleocytosis can reflect post-neurosurgical chemical meningitis or intraventricular drug irritation; such patterns mandate close correlation with the operative course and therapies.

Taken together, vCSF cytology is more susceptible to confounding by blood contamination and device-related foreign-body-reaction, and more prone to “false blandness” from spatial gradients than lumbar CSF. The safest approach to correct interpretation includes explicit site-labelling, trend-based interpretation, and tight integration with clinical findings, microbiology, and imaging.

## Staying up to date: emerging tools for CSF cytopathology in the era of digitalization and Artificial Intelligence (AI)

Currently, new methods such as liquid biopsies and high-dimensional immunophenotyping are developing at an ever-increasing pace. Therefore, it may be justified to ask whether classical CSF cytopathology, or indeed conventional CSF diagnostics in general, will become less important soon. However, it should be noted that there are also significant and promising developments in cytopathological diagnostics that will likely underpin the continued importance of traditional cytopathology, as a basis of, and addition to, new powerful techniques.

### Artificial intelligence in CSF and body-fluid cytopathology: current state and prospects

Over the past five years, deep-learning (DL) systems have moved from early proof-of-concept approaches to small clinical feasibility studies. In CSF, convolutional models trained on digitized cytospins can perform five-way leukocyte and tumour-cell differentials and even subtype carcinoma cells by putative primary site [52, 114, 140]. A 2022 five-way cell classification model pipeline reported robust detection of cancer cells and even robust cancer subtype classification on > 50k single-cell crops from 90 patients with leptomeningeal metastasis, while separate groups demonstrated explainable DL that classifies diagnostically relevant CSF cell-types and provides case-level labels on whole-slide images [140]. Early neural-network approaches have also tackled pre-screen tasks such as cell counting and coarse lineage gating to prioritize fields of view for human evaluation, which seems especially promising in low-cellularity specimens typical of CSF [52]. Although encouraging, these studies remain single centre, often with curated datasets and limited external validation, so generalizability across labs, stains, and scanners is not yet assured.

By now, evidence is stronger in the cytology of other body-fluids than CSF, especially pleural effusions, where AI application on whole-slide images has reached diagnostic-grade performance for malignancy detection and class discrimination in retrospective cohorts [91]. Recent narrative, and systematic reviews conclude that AI can augment but not replace expert assessment, emphasizing the need for multi-site validation and workflow integration [16, 60].

Importantly, regulated AI triage is already a reality in cytology: the Hologic Genius™ Digital Diagnostics platform has FDA clearance (DEN210035) for pap smears, where AI pre-screens and surfaces regions of interest before pathologist sign-out. This provides a regulatory precedent demonstrating that AI-assisted image triage in cytology is feasible and acceptable, a framework that may become transferable to body-fluid cytology as digital image acquisition, data standardization, and model validation continue to mature. Early translational work also explores imaging-flow-cytometry in combination with AI application for malignant-cell enrichment in effusions, hinting at hybrid pipelines that hand off AI-flagged candidates to immunocytochemistry or liquid-biopsy for confirmation and typing [71, 76, 84]. Practical near-term use scenarios for CSF laboratories may therefore be AI-assisted pre-screening to rank slides/regions with suspected tumour cells or atypia for early human review, quality control support (flagging out-of-distribution stains, focus, or improbable differentials), and decision support in equivocal cases by visual explanations that point to cells driving a “malignant-suspicious” score; at this point in time this remains always under the oversight of a trained cytopathologist. What will be needed next are prospective, multi-centre studies using vendor-agnostic scanners, standardised staining, and external test sets, with reporting of calibration, class-imbalance handling, and human-factors outcomes (human time saved, inter-observer agreement).

### Telecytology for CSF: rationale, validation, workflows, and limits

Telemedicine approaches, specifically with regards to (cyto-)pathology, have emerged to maintain wide-spread and timely expert input while minimizing transfers and turnaround delays.

For cytopathology in general, contemporary digital pathology guidance supports validation for cytology, including minimum case numbers, documentation of concordance to glass, and ongoing quality checks [3, 33]. Telecytology can be delivered via remote-controlled live microscopy (camera-equipped microscope with secure, low-latency streaming) or whole-slide imaging (WSI) for store-and-forward consultation. Routine whole-slide digitization of CSF cytospins may, however, be technically challenging, particularly in paucicellular specimens, because automated cell detection and autofocus algorithms may fail to identify sparsely distributed cellular material or an optimal focal plane [30, 68]. Practical approaches adopted from general cytopathology include manual definition of the cellular deposition area and additional focus points as well as multiplane or Z-stack scanning, or extended-depth-of-field acquisition where available [30, 68]. These approaches can improve visualization of cellular material distributed across different focal planes, although at the expense of longer scanning times and larger file sizes. Importantly, these strategies are largely derived from experience with cytological specimens in general and have not yet been systematically validated specifically for CSF cytospins. Prospective studies in cytopathology (including body-fluid preparations and rapid on-site evaluation (ROSE) workflows) show high concordance for adequacy calls and preliminary diagnoses when readers are trained and infrastructure is stable [12, 123].

In CSF cytopathology, although not yet widely adopted, telecytology has considerable potential: under growing economic pressures and service consolidation for hospitals and laboratories, it offers a practical way to centralise expertise and facilitate access to expert cytopathological assessment, particularly at peripheral sites without local expertise. Establishing regional tele-networks would require centres and peripheral sites to join forces and agree on appropriate standards for joint diagnostics (e.g. sending standardised, prepared, fixed cytospin slides to expert centres or on-site microscopy with tele-consultation). Maintaining high standards in pre-analysis, slide preparation, and staining may be more difficult in decentralised facilities, when there is no direct local feedback on quality aspects.

## Summary

Taken together, CSF cytology has evolved to be and will remain a powerful tool in CSF analysis. Correlation of cytology findings with clinical data, and integration of results along with protein findings in an integrated report, are crucial. Expert microscopic assessment remains the reference standard for qualitative CSF cytology. Yet, computer-assisted technologies are rapidly approaching and hold considerable potential. Their implementation should be accompanied by critical evaluation and validation to ensure diagnostic accuracy as technological and organizational changes in CSF diagnostics unfold over the coming decade.

## Supplementary Information

Below is the link to the electronic supplementary material.

Supplementary file1 (DOCX 41 KB)

## Data Availability

No new data were generated or analysed in support of this review. Data sharing is therefore not applicable to this article.
