# Supplementary material for: CSF cytology: core principles, diagnostic pitfalls and emerging digital tools including artificial intelligence and telecytology
Source: Acta Neuropathol. 2026 Oct 30;152(1):64. doi: 10.1007/s00401-026-03101-7 (PMC13722749; doi:10.1007/s00401-026-03101-7)
Supplement: Supplementary file 1 [file 401_2026_3101_MOESM1_ESM.docx]

**Supplementary Table S1 Comparative studies of automated versus manual cerebrospinal fluid cell counting**

Characteristics and principal findings of original human studies identified in an update search of PubMed and the Cochrane Library (1 January 2019 to 19 September 2026), supplemented by backward and forward citation screening. Eligible studies directly compared an automated or semi-automated platform with manual chamber counting or optical microscopy for quantitative cerebrospinal fluid cell analysis. Studies already included in Waldrop et al. [16], automated-versus-automated comparisons, case reports, and studies without extractable CSF-specific quantitative results were excluded. Mixed-body-fluid studies were retained when a distinct CSF subgroup was reported. Sample sizes refer to the CSF subgroup unless otherwise stated.

| **Study** | **CSF samples** | **Automated or semi-automated platform** | **Manual reference method** | **Evaluated parameters** | **Principal findings** | **Key limitations / interpretation** |
| --- | --- | --- | --- | --- | --- | --- |
| **Buoro et al. 2019 [3]** | 132 CSF (351 BF total) | Mindray BC-6800 BF mode; Sysmex XN-series BF mode | Nageotte or Buerker chamber; cytospin morphology | TC, WBC, MN, PMN, neutrophils, EO and HF-BF | At the analyser-specific CSF cut-offs (XN WBC ≥5 cells/µL; BC WBC ≥4 cells/µL), between-analyser agreement was high (κ 0.87). Significant analyser-dependent bias was present, especially for HF-BF and EO-BF. | Mixed-fluid adult cohort. BC-BF could not classify cells in 81/132 CSF samples with TC ≤ 5 cells/µL; instrument-specific cut-offs and morphological review remain necessary. |
| **Maleb et al. 2020 [11]** | 93 CSF | Sysmex UF-1000i BF mode | Fast-Read 102 disposable counting chamber | WBC and RBC counts | Concordance was 99.06% for WBC and 85.18% for RBC. Passing-Bablok slopes indicated positive proportional bias (WBC 1.16; RBC 1.18); median WBC was 6 cells/µL manually versus 9 cells/µL on UF-1000i. | Only 13 samples had manual WBC <5 cells/µL. Bacteria, yeast, debris and incompletely lysed RBC may be misclassified; the authors regard microscopy and automation as complementary. |
| **Cho et al. 2020 [4]** | 88 CSF (542 BF total) | Beckman Coulter DxH 800; Sysmex XN-350; Sysmex UF-5000 | Neubauer haemocytometer; cytospin differential | TNC, RBC, MN, PMN; HF-BF reflex testing | Good agreement for TNC and RBC counts, except for CSF RBC counts on the DxH 800. Differential counts were more variable; reflex rules for HF-BF and eosinophils were proposed. | Mixed-fluid study; automated differential and malignant-cell screening required manual confirmation. |
| **Lee et al. 2021 [9]** | 63 CSF (165 BF total) | Sysmex XN-350 BF mode | Manual chamber counting | TC, WBC, RBC and differential counts | Acceptable analytical performance; WBC LoD 1 cell/µL and LoQ 5 cells/µL. CSF differential counting, particularly monocyte/mononuclear measurements, showed weaker agreement. | Counts around the LoQ and CSF differential counts require manual verification. |
| **Lu et al. 2021 [10]** | 220 CSF | Compact automated fluorescence microscope with preloaded dry-reagent chamber | Reusable glass haemocytometer; acetic-acid preparation for WBC classification | WBC and RBC counts; exploratory WBC differentiation | All automated low-count results were manually normal (192/192) and all high-count results abnormal (18/18); 10/220 (4.5%) intermediate results required manual review. The 20% CV LoQ was about 6 cells/µL for WBC and RBC. | Proof-of-concept with few abnormal samples, 1-10 h delay after the clinical count, systematic RBC undercounting at high concentrations and poor differential agreement in 21 samples (Pearson r=0.66). |
| **Siatkowski et al. 2022 [15]** | 54 CSF; low-count analyses pooled with 8 CAPD samples (223 BF total) | Sysmex UF-4000 BF mode | KOVA chamber light microscopy; cytospin/MGG differential | WBC and RBC counts | In pooled CSF/CAPD samples, agreement was excellent for WBC (R²=0.9984; bias -1 cell/µL) and RBC (R²=0.9966; bias -43 cells/µL). Sensitivity and specificity were both 100% at 5 WBC/µL. | CSF and CAPD were pooled because of small subgroups; estimates were therefore not CSF-specific. Uncertainty at the 5 WBC/µL cut-off was 20%, and atypical/non-haematopoietic cells were not assessed. |
| **Kalambi-Matengu et al. 2022 [7]** | 46 CSF | Siemens ADVIA 2120i CSF module | Mean of two counts using an improved Neubauer chamber | RBC, WBC, PMN and mononuclear/lymphocyte counts | Acceptable precision, linearity and carryover. RBC and PMN counts showed good accuracy; the lymphocyte result had an overall positive bias of 7.5 cells/µL. | Small single-centre cohort; 300 µL sample volume required; no dedicated morphology flagging. |
| **Baran and Ilki 2023 [1]** | 433 CSF (634 BF total) | Sysmex UF-5000 BF mode | Thoma chamber microscopy at ×400; Gram stain and culture | WBC and bacterial counts | CSF WBC counts showed categorical Spearman correlation of 0.70 (p<0.005) across five concentration bands. Automated bacterial and WBC counts were higher in culture-positive samples; 38/433 CSF cultures were positive. | Broad categories rather than continuous agreement metrics; heterogeneous adult/paediatric cohort. Reported CSF sensitivity was 56% in adults and 67% in children; no differential counts and 600 µL required. |
| **Evren et al. 2023 [5]** | 78 CSF (200 BF total) | Sysmex XN-1000 BF mode | Bright-lined Neubauer chamber; Wright-Giemsa differential | WBC, PMN and MN counts | For CSF WBC, r=0.96, Passing-Bablok slope 1.13 and mean absolute difference -17.3 cells/µL. Differential correlations were lower (PMN r=0.75; MN r=0.72). | CSF samples had relatively high cellularity; performance near the normal reference range could not be established. |
| **Ren et al. 2023 [14]** | 40 CSF | Sysmex XN-9000 BF mode | Improved Neubauer chamber; optical microscopy after one- or two-step cytocentrifugation | RBC, WBC, PMN and MN counts; morphology | Correlation with microscopy was excellent for RBC (R²=0.9989), WBC (R²=0.9637) and PMN (R²=0.98), but lower for MN (R²=0.70). Two-step cytocentrifugation recovered more cells than one-step preparation. | Small, selected cohort. HF-BF did not reliably identify tumour cells, phagocytes, plasma cells or leukaemia cells; detailed morphological review remained mandatory. |
| **Bensalah et al. 2024 [2]** | 121 CSF | Sysmex XN-1000 BF mode | Fast-Read 102 chamber; counts by at least two experienced haematologists | WBC and RBC counts | Passing-Bablok and Bland-Altman analyses did not demonstrate interchangeability or concordance between methods. | Acute-leukaemia population with disease-specific cellular patterns; findings may not generalise to routine neurological CSF. |
| **Park et al. 2024 [13]** | 150 clinical CSF plus artificial samples | Microscanner C3 automated image-based cell counter | Neubauer chamber; flow cytometry as an additional comparator | WBC and RBC counts | Lowest detectable counts were 3.33 WBC/µL and 3.67 RBC/µL. Correlation with the Neubauer chamber was R=0.9377 for WBC and R=0.9952 for RBC; precision exceeded manual counting. | Single-centre evaluation of a newer image-based platform; external validation is limited. |
| **Haarmann et al. 2025 [6]** | 119 CSF (64 lumbar puncture; 55 EVD) | Sysmex XN-9000 BF mode | Unstained Fuchs-Rosenthal chamber count | Total CSF leukocyte count | Strong overall agreement. In 59 samples with <20 cells/µL, correlation remained high (R=0.90); two paired results crossed the 5-cells/µL clinical threshold. | Short single-centre sampling period; threshold discordance, although uncommon, can be clinically relevant. |
| **Oprea et al. 2026 [12]** | 77 CSF | UriSed Mini semi-automated image-based analyser | Counts by two technicians using Fuchs-Rosenthal chambers | Quantitative and categorical nucleated-cell counts | Mean quantitative difference was 0.6 cells/µL and categorical agreement was excellent (weighted κ 0.96, 95% CI 0.89-1.00). Analytical performance met prespecified requirements. | Single-centre cohort; broader external validation of the CSF software is still required. |
| **Kukrálová et al. 2026 [8]** | 89 CSF | Sysmex XN-1000 BF mode | Fuchs-Rosenthal chamber | MN, PMN and RBC counts; oligocytosis/pleocytosis classification | No significant difference for MN or RBC counts; PMN counts differed (p=0.0002). Agreement for oligocytosis versus pleocytosis was very good (κ 0.89). | PMN misclassification occurred particularly in siderophage-rich neonatal samples; manual verification remains necessary. |

**Notes**

- The table represents a targeted update to the evidence base summarised by Waldrop et al. [16].
- A mixed-body-fluid study was included only when CSF was analysed as a distinct subgroup. Pooled estimates are explicitly identified.
- Counts, coefficients and diagnostic performance measures are reproduced as reported by the study authors. Definitions and cut-offs differed between studies.

**Abbreviations**

BC, Mindray BC-6800 analyser; BC-BF, Mindray BC-6800 body-fluid mode; BF, body fluid; CAPD, continuous ambulatory peritoneal dialysis; CI, confidence interval; CSF, cerebrospinal fluid; CV, coefficient of variation; EO, eosinophils; EO-BF, eosinophils counted in body-fluid mode; EVD, external ventricular drain; HF-BF, high-fluorescence body-fluid cells; LoD, limit of detection; LoQ, limit of quantification; MGG, May-Grünwald-Giemsa; MN, mononuclear cells; PMN, polymorphonuclear cells; RBC, red blood cells; TC, total cells; TNC, total nucleated cells; WBC, white blood cells; XN, Sysmex XN-series analyser.

**References**

1. Baran E, Ilki AA (2023) Evaluation of Sysmex-UF 5000-BF module for sterile body fluids. An alternative for conventional methods? Clin Lab 69:706–713. https://doi.org/10.7754/Clin.Lab.2022.220522
2. Bensalah M, Lekfif H, Berhili A, Khermach A, Seddik R (2024) Comparative study of cerebrospinal fluid evaluation in patients with acute leukemia using the manual method and Sysmex XN-1000. Cureus 16:e72423. https://doi.org/10.7759/cureus.72423
3. Buoro S, Seghezzi M, Dominoni P, Moioli V, Manenti B, Previtali G et al (2019) Lack of harmonization in high fluorescent cell automated counts with body fluids mode in ascitic, pleural, synovial, and cerebrospinal fluids. Int J Lab Hematol 41:277–286. https://doi.org/10.1111/ijlh.12968
4. Cho J, Oh J, Lee SG, Lee YH, Song J, Kim JH (2020) Performance evaluation of body fluid cellular analysis using the Beckman Coulter UniCel DxH 800, Sysmex XN-350, and UF-5000 automated cellular analyzers. Ann Lab Med 40:122–130. https://doi.org/10.3343/alm.2020.40.2.122
5. Evren E, Us E, Hekimoğlu CH, Karahan ZC (2023) Cell counting chamber vs. Sysmex XN-1000 for determining white blood cell count and differentiation for body fluids. Turk J Biochem 48:19–25. https://doi.org/10.1515/tjb-2022-0179
6. Haarmann A, Schubert J, Steigerwald U, Schuhmann MK (2025) Automated cell counting in CSF diagnostics revisited—friend or foe? Diagnostics (Basel) 15:1202. https://doi.org/10.3390/diagnostics15101202
7. Kalambi-Matengu EN, Coovadia Y, Haupt L (2022) Validation of the cerebrospinal fluid module of the Siemens ADVIA 2120i for automated cell counts of cerebrospinal fluid. J Med Lab Sci Technol S Afr 4:78–83. https://doi.org/10.36303/jmlstsa.111
8. Kukrálová S, Brož P, Černá J, Koldušková K, Naušová E, Ženková J et al (2026) Comparison of quantitative cerebrospinal fluid cytology using manual counting and the Sysmex XN-1000. Bratisl Med J 127:323–329. https://doi.org/10.1007/s44411-025-00425-w
9. Lee J, Cho Y, Kim HS, Kang HJ, Kim M, Lee YK (2021) A comparison of the analysis of 3 types of body fluids using the XN-350 hematology analyzer versus light microscopy assessment. Medicine (Baltimore) 100:e24852. https://doi.org/10.1097/MD.0000000000024852
10. Lu Q, Chu K, Dou H, Smith ZJ (2021) A sample-preparation-free, automated, sample-to-answer system for cell counting in human body fluids. Anal Bioanal Chem 413:5025–5035. https://doi.org/10.1007/s00216-021-03466-6
11. Maleb A, Bouayadi O, El Malki J, Rifai S, Lamrabat S, Benaissa E et al (2020) Cytological examination of cerebrospinal fluid: Sysmex UF-1000i versus optical microscopy. Anal Biochem 589:113501. https://doi.org/10.1016/j.ab.2019.113501
12. Oprea OR, Fotache AM, Mănescu IB, Dobreanu M (2026) Evaluation of analytical performance and clinical applicability of the UriSed Mini, a semi-automated urine analyzer for cell counting in cerebrospinal fluid. Biochem Med (Zagreb) 36:020705. https://doi.org/10.11613/BM.2026.020705
13. Park I, Choi M, Lee E, Park S, Jang WS, Lim CS et al (2024) Evaluation of the Microscanner C3 for automated cell counting in cerebrospinal fluid analysis. Diagnostics (Basel) 14:2224. https://doi.org/10.3390/diagnostics14192224
14. Ren XQ, Jiang X, Pi YQ, Wu ZK, Jiang HB, Wang HC (2023) Comparison of cerebrospinal fluid cellular analysis by optical microscopy and Sysmex XN 9000. Clin Lab 69:2274–2279. https://doi.org/10.7754/Clin.Lab.2023.230403
15. Siatkowski M, Dahyot S, Pestel-Caron M, Boyer S (2022) Performance evaluation of UF-4000 body fluid mode for automated body fluid cell counting. Clin Chim Acta 531:152–156. https://doi.org/10.1016/j.cca.2022.04.002
16. Waldrop GE, Cocuzzo K, Schneider CL, Kim CY, Goetz TG, Chomba MS et al (2024) Accuracy of automated analyzers for the estimation of CSF cell counts: a systematic review and meta-analysis. Int J Lab Hematol 46:234–242. https://doi.org/10.1111/ijlh.14236
